# Genetic variability and structure of the water vole *Arvicola amphibius* across four metapopulations in northern Norway

**DOI:** 10.1002/ece3.499

**Published:** 2013-02-18

**Authors:** Claudia Melis, Åsa Alexandra Borg, Henrik Jensen, Eirin Bjørkvoll, Thor H Ringsby, Bernt-Erik Sæther

**Affiliations:** 1 Department of Biology, Centre for Conservation Biology, Norwegian University of Science and TechnologyTrondheim, N-7491, Norway; 2 The Norwegian Directorate for Nature ManagementPO Box 5672 Sluppen, Trondheim, N-7485, Norway

**Keywords:** Dispersal, genetic structure, isolation by distance, microsatellite, patchy habitat

## Abstract

Water vole *Arvicola amphibius* populations have recently experienced severe decline in several European countries as a consequence of both reduction in suitable habitat and the establishment of the alien predator American mink *Neovison vison*. We used DNA microsatellite markers to describe the genetic structure of 14 island populations of water vole off the coast of northern Norway. We looked at intra- and inter-population levels of genetic variation and examined the effect of distance among pairs of populations on genetic differentiation (isolation by distance). We found a high level of genetic differentiation (measured by *F*_ST_) among populations overall as well as between all pairs of populations. The genetic differentiation between populations was positively correlated with geographic distance between them. A clustering analysis grouped individuals into 7 distinct clusters and showed the presence of 3 immigrants among them. Our results suggest a small geographic scale for evolutionary and population dynamic processes in our water vole populations.

## Introduction

The amount of genetic variation within a population has implications for its ability to cope in the face of environmental changes (Wright 1978), such as habitat fragmentation or loss, introduction of new predators, competitors, or pathogens, as well as climate change. Habitat loss and fragmentation are one of the main threats to biodiversity because they usually result in reduced population size and viability (see Fahrig 2003 for a review). During the process of fragmentation, populations are likely to go through bottlenecks reducing the amount of genetic variation (e.g. Gibbs 2001). In small populations, random genetic drift is further reducing genetic variation, and renders these populations more susceptible to inbreeding depression and extinction (e.g. Nei et al. 1975).

**Figure 1:**
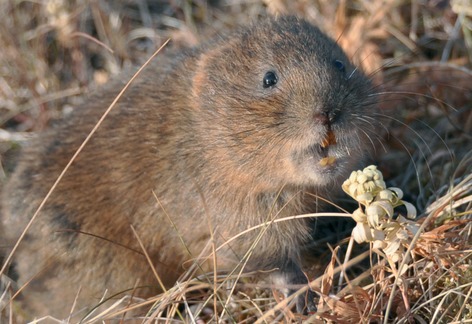
Adult individual of water vole marked at Sleneset (northern Norway).

A metapopulation is a set of populations that occupy a series of discontinuous habitats connected by limited migration (Hanski and Simberloff 1997). The persistence of a metapopulation is assured by the balance between extinction and recolonization of these habitat patches by means of dispersal (Levins 1969; Harrison and Taylor 1997; Hanski 1999). As such, metapopulations are well-suited model systems to study the consequences of habitat fragmentation and small population size. Although there is still lack of consensus about the real existence of metapopulation structures in small mammals, black-tailed prairie dogs *Cynomys ludovicianus*, American pika *Ochotona princeps,* and water vole *Arvicola amphibius* (formerly named *Arvicola terrestris*) seem to best conform to the metapopulation paradigm (Lambin et al. 2004). The latter has also been defined as an ideal species to study the genetic structure of vertebrate populations in patchy environments because they occur in naturally patchy habitats (Aars et al. 2006). The water vole (Fig. 1) is a Eurasian species cataloged as a “least concern” species by the IUCN Red List. They are rather large rodents (200–300 g), typically associated with riparian habitats, and have in the last 50 years gone through a dramatic decline in several European countries (Saucy 1999). This has been attributed to both habitat loss and the introduction and spread of the alien American mink *Neovison vison*, which is a very efficient predator of water voles, being able to enter their burrow systems (Barreto and Macdonald 2000). Water vole populations, like many rodent species in Fennoscandia, are likely to experience cyclic population declines. In addition, each year, the colonies go through a period of growth in summer, replaced by drastic reduction in number with few individuals surviving the winter (about 10%, according to the recapture rate of individuals, which survived the winter and individuals born in the same year of capture, Melis et al. unpubl. data). These surviving individuals will produce several offspring in spring–summer, which will again go through a reduction in numbers the following winter. Such dynamics is expected to have a profound impact on population structure and genetic variability (Wright 1978), because of loss of allele diversity and increase in homozygosity that can lead to inbreeding depression (e.g. Nei et al. 1975). When such populations are located on islands, and especially when the populations are small, the level of dispersal is therefore expected to be a very important factor in shaping genetic structure and variation (Telfer et al. 2003). In our study area in northern Norway, water vole populations consist of colonies occupying islands and, because the colonies go through extinction and recolonization events caused by dispersal that maintain a viable total population, their structure fits well with Levin's metapopulation theory.

Although water voles are currently not listed as threatened in Norway, the American mink has established throughout Norway with the exception of some insular areas (Bevanger 2007) and this alien predator is thus likely to represent a serious threat to the species in Norway, such as happened in other European populations (see Bonesi and Palazon 2007 for a review). Moreover, in our study area, the water vole is a very important prey for the endangered eagle owl *Bubo bubo* (constituting 92% of the diet in frequency of occurrence, Melis et al. unpubl. data). In one of our studied metapopulations, at Sleneset, the eagle owl has one of the highest breeding densities reported for the whole of Europe (Jacobsen and Røv 2007). It is also possible that, similar to other digging rodents (e.g. Zhang et al. 2003), water voles act as ecosystem engineers and promote diversity. If so, they may modify soil composition by aerating and mixing the soil and therefore increasing its water-holding capacity and slowing down erosion, and also act as disturbance on plant communities by selectively feeding on them.

Previous studies on water vole populations in Scotland showed high levels of genetic diversity and that the genetic structure of colonies in this species often departed from equilibrium, which is often assumed in gene flow studies (Stewart et al. 1999). Another study conducted in Scotland found that water vole populations living on small islands retained a lower genetic variability and were more differentiated between them with respect to mainland populations (Telfer et al. 2003). A study conducted in France on water vole populations in mainland showed a very high within-populations genetic diversity, which was not directly related to abundance at the time of sampling, and a very clear spatial structure in genetic diversity, which was consistent with a spatially restricted dispersal (Berthier et al. 2005). A comparison between fragmented populations living in patchy habitats and continuous ones revealed comparable values of genetic variability, which could be either consequence of effective long-distance dispersal or of low variance in reproductive success among females (Aars et al. 2006).

In this study, we used DNA microsatellite markers to describe the genetic structure of 14 island populations of water vole. We looked at intra- and inter-population levels of genetic variation and examined the effect of distance among all pairs of populations on genetic population structure (isolation by distance, Slatkin 1993). In addition, we identified likely immigrant individuals based on their microsatellite profile to estimate levels of gene flow.

## Methods

### Study area

**Figure 2:**
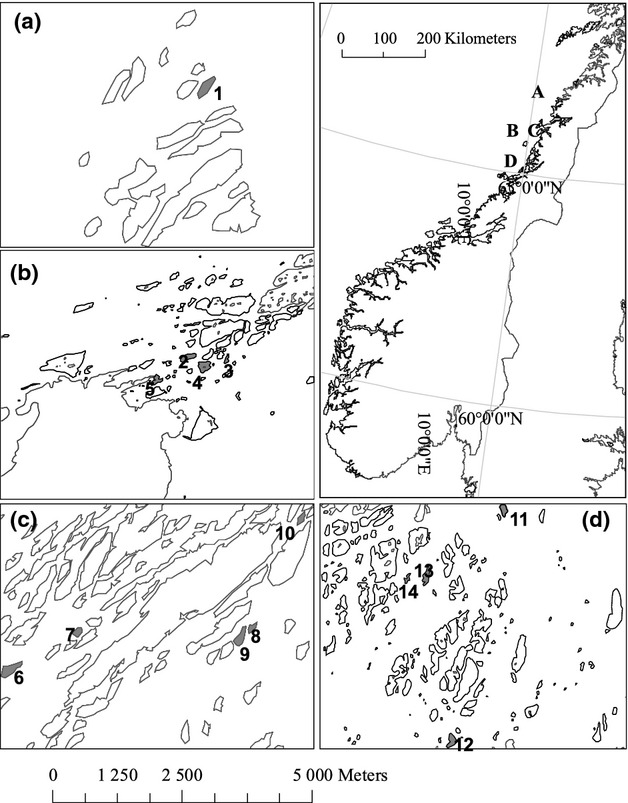
Study area at the coast of northern Norway where the genetic structure of water vole was investigated in 2006. The sampled island populations are indicated by numbers (1–14) and the localities by letters (a: Myken; b: Lovund; c: Sleneset; d: Lånan). The scale bar at the bottom refers to the scale in maps showing details of the 4 localities.

The study was conducted in June–September 2006 on 14 uninhabited treeless small islands with sparse grass cover (some dominated by meadowsweet *Filipendula ulmaria* others dominated by crowberry *Empetrum nigrum*, cloudberry *Rubus chamaemorus* and common juniper *Juniperus communis*) along the coast of northern Norway (from 65.86°N to 67.77°N, Fig. 2, Table 1). Several of the smallest islands that were surveyed did not have water voles in 2006 and were therefore not sampled, although they showed presence of animals in subsequent years (data not shown here), which confirmed the extinction and recolonization dynamics in these populations. Due to the limited duration of the study, we were not able to document the existence of multiannual cycles in this species; however, local people reported years with exceptional abundance of water voles followed by years when it was very difficult to observe animals, so it is likely that these water voles also go through multiannual cycles.

**Table 1:** Sampling localities, geographic coordinates of the islands, island area, *N* number of individual water voles sampled and genotyped at 13 microsatellite loci in each population in northern Norway, and basic population-level statistics of genetic variability: *N* sample size, *N*_cmr_ mean population size estimated by capture-mark-recapture methods. *A*_R_ allelic richness corrected for minimum sample size, *H*_O_ observed heterozygosity, *H*_E_ expected heterozygosity*, F*_IS_ inbreeding coefficient, HW level of significance (Bonferroni-adjusted 5% level of significance: *P* = 0.0036, significant values in bold) for test of deviance from Hardy–Weinberg equilibrium across all loci. None of the *F*_IS_ was significantly different from zero when correcting the *P* value for the number of simultaneous tests (adjusted 5% level of significance: *P* = 0.0003)

Locality	Island nr.	North	East	Area (m^2^)	*N*	*N*_cmr_	*A*_R_	*H*_O_	*H*_E_	*F*_IS_	HW
Myken	1	66.770	12.475	35000	15	30	1.305	0.164	0.129	−0.280	0.492
Lovund	2	66.376	12.368	18100	7	7	2.119	0.571	0.450	−0.300	0.977
	3	66.377	12.382	6550	5	6	1.540	0.369	0.253	−0.548	0.538
	4	66.377	12.381	7050	4	-*	1.632	0.374	0.252	−0.416	0.990
	5	66.373	12.359	12330	52	53	2.234	0.463	0.486	0.046	**0.001**
Sleneset	6	66.324	12.520	11800	8	9	2.314	0.442	0.485	0.094	0.792
	7	66.335	12.541	9150	6	-*	2.234	0.577	0.484	−0.216	1.000
	8	66.334	12.617	19900	4	5	1.684	0.308	0.272	−0.157	0.986
	9	66.332	12.613	34900	115	115	1.922	0.384	0.387	0.007	0.019
	10	66.351	12.632	15200	76	85	2.002	0.418	0.412	−0.013	0.240
Lånan	11	65.884	11.830	8350	4	-*	1.283	0.153	0.115	−0.412	0.879
	12	65.864	11.820	8370	17	18	1.572	0.236	0.220	−0.071	0.718
	13	65.878	11.813	10150	7	-*	1.410	0.132	0.177	0.273	0.480
	14	65.877	11.809	2350	3	3	1.769	0.256	0.261	0.024	1.000
				Total	323						

* Too large confidence intervals.

The breeding season of this species in our study area extends from April to August (Frafjord personal communication). The islands are geographically clustered in 4 groups of islands among which we might expect little dispersal to occur, from south to north: Lånan (*N* = 4), Slesenet (*N* = 5), Lovund (*N* = 4), and Myken (*N* = 1). A higher level of dispersal is expected to occur within each of these groups of islands.

Geographic population coordinates, island size, and sampling information are shown in Table 1. A matrix showing pairwise geographic distances between the centers of island populations is presented in Appendix 1.

### Data collection

Individual water voles were captured-mark-recaptured by means of a grid of Sherman foldable traps baited with fresh carrots and filled with dry grass as bedding material. The same type of grid and trapping effort were used on all islands (see below). Accordingly, we believe that the sample sizes reflect real differences in population sizes and are not an artifact due to differences in sampling effort. Population size for each population was also estimated with the software MARK 6.1 (White and Burnham 1999). We assumed that within each trapping session, the population was closed, and we selected the closed capture Huggins estimator (Huggins 1989). We compared four models that differed in their assumed sources of variation in probability of capture (*pi*) and probability of recapture (*ci*). Model M_0_, assumes equal capture probability for all animals on all trapping occasions; M_b_ assumes different capture and recapture probabilities; M_*t*_ assumes that capture probability varies with time; M_h_ assumes different capture probabilities for different individuals. The minimum adequate model was selected by comparing the Akaike Information Criterion corrected for small sample sizes (AICc, Burnham and Anderson 2002) and varied among islands. The number of individuals sampled and the estimated size of the population were highly correlated (*r*_p_ = 0.992, *P* < 0.001, *N* = 10, Table 1).

However, for four islands, the confidence intervals were too wide to provide reliable estimates (Table 1). This was due to estimation problems when very few new individuals were marked at the end of the trapping session. Traps were placed on a 20-m cell grid covering the part of the island with soil and vegetation, thus excluding rocky areas (*N* of traps on each island = 20–80). Each capture session continued until we reached a 40% cumulative recapture rate (*N* individuals recaptured/*N* individuals captured). The longest session lasted 10 days. The traps were checked at least twice a day, but more often when the weather was very cold or very warm to prevent any negative effects of trapping on the population. About 40% of the capture consisted of individual classified as “adults” according to their size and 43.7% of the capture were males. For details about the capture and measurement methods, see Melis et al. (2011). At first capture, a tissue sample was taken from the ear by a 2-mm biopsy punch (BC-BI-0500 Stiefel Biopsy Punch) and the individuals were marked with a passive integrated transponder tag inserted by injection under the skin of the neck (Trovan ID100, 2.12 × 11.5 mm, Trovan Ltd., Douglas, U.K.). The individuals were subsequently released at the exact location of capture. The tissue samples were stored in 96% ethanol at −20°C and DNA was later extracted using a vacuum extraction method following the procedure outlined in Elphinstone et al. (2003) and stored at −32°C until required for PCR.

### Microsatellite genotyping

DNA from the samples reported in Table 1 was analyzed by PCR-amplification of 13 microsatellite loci in two multiplex panels: panel 1, including AV3, AV8, AV9, AV10, AV11, AV12, AV14 (Berthier et al. 2005) and panel 2, including AT2, AT9, AT13, AT22, AT24, and AT25 (Berthier et al. 2004). SRY-HMG (included in panel 2) was used for sex determination (Bryja and Konečny 2003) because juveniles have little sexual dimorphism in this species. For panel 1, we labeled the forward (F) primers with the same fluorescent dyes as Berthier et al. (2005), while for panel 2, we used FAM dye for all the F primers because products did not overlap in size.

All the individuals were genotyped at the 14 loci in 10 μL reactions. Each reaction contained 2 μL primer-mix, 5 μL Qiagen multiplex PCR solution (containing *Taq*-polymerase, dNTPs and PCR buffer) and 3 μL DNA (approx. 20 ng/μL). The final concentration of each F and reverse (R) primer in the multiplex PCR was 0.0625 μmol/L. The touchdown PCR profile had an initial denaturation step at 94°C for 15 min, then 12 cycles starting with 30 sec at 94°C and 1 min 30 sec at temperature 62–50°C, decreasing 1 degree pr cycle, and an extension step at 72°C for 1 min. This was followed by 23 cycles with 30 sec at 94°C, 50°C for 1 min 30 sec, and 72°C for 1 min. The PCR ended with an elongation step at 60°C for 5 min and then the PCR products were stored at 4°C. After amplification, the PCR products were diluted 1:2 by adding 10 μL ddH_2_O to the products. 1 μL of the diluted PCR product was then mixed with 10 μL of a mix of Hi-Di formamide (Applied Biosystems Inc., Foster City, CA) and GeneScan 600 LIZ (Applied Biosystems) size standard (for one sample: 9.524 μL Hi-Di formamide and 0.476 μL 600 LIZ). Electrophoresis and separation of alleles (fragment analysis) were conducted on an ABI3130xl Genetic Analyzer (Applied Biosystems). Individual alleles on each microsatellite locus were scored using the software GeneMapper 4.0 (Applied Biosystems).

### Software and statistics

The number of alleles (*n*_*a*_), allelic richness corrected for minimum sample size (*A*_R_), observed (*H*_O_) and expected (*H*_E_) heterozygosities for each locus, as well as inbreeding coefficients (*F*_IS_) for each population and overall *F*_ST_, were calculated in FSTAT version 2.9.3.2 (Goudet 1995). We tested for departure from Hardy–Weinberg equilibrium (HW) and linkage disequilibrium (LD) using exact tests based on a Markov chain algorithm implemented in the program GENEPOP 3.4 (Raymond and Rousset 1995). Evidence for scoring error due to stuttering, large allele dropout and null alleles was checked with the software Microchecker 2.2.3 (Van Oosterhout et al. 2004).

We compared pairwise genetic distances to pairwise geographic distances (Appendix 1) to investigate isolation by distance among populations (Slatkin 1993). The computer program GENEPOP 3.4 (Raymond and Rousset 1995) was used to estimate pair-wise *F*_ST_ (Weir and Cockerham 1984) among the sampled populations (see Appendix 1). The estimates from these analyses were further analyzed and related to geographic distance, using the software R (R Development core team 2006). The R package ECODIST (Goslee and Urban 2007) was used to carry out Mantel tests (Mantel 1967), which allow for inter-dependence of data points in the analyses (e.g. Underwood 1997). In the Mantel tests, a Pearson's rank correlation coefficient was calculated and statistical significance was estimated by 1000 permutations.

Because the study area is two-dimensional, we used the transformed *F*_ST_ (i.e. 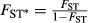) and decimal logarithm of geographic distance in the analyses (see Rousset 1997).

The R package HIERFSTAT (Goudet 2005) was used to determine the relevant unit of population structure according to two hierarchical levels: island and locality. The significance of these different levels was tested with a G-based randomization test implemented in the package and the number of randomization was set to 10,000.

STRUCTURE 2.3.3 (Pritchard et al. 2000; Pritchard and Wen 2003) was used to estimate the spatial structure of the genetic data. STRUCTURE considers multilocus genotypes and attempts to minimize linkage disequilibrium and Hardy–Weinberg disequilibrium by estimating the number of populations (*K*) on the basis of individual data. In STRUCTURE, we ran five iterations for each *K* = 1–20 (100,000 burn-in period length, 500,000 Monte Carlo repetitions) using the admixture model, correlated allele frequencies and no prior information of the sampling locality. Furthermore, we followed the procedure described by Evanno et al. (2005) to identify the principal hierarchical level of structure in our data. Clustering was performed and the individuals were assigned to groups using *q*-values. To detect the number of first generation immigrants among the 7 clusters of islands estimated by STRUCTURE, we used the software Geneclass 2.0 (Piry et al. 2004) and we followed the settings recommended when not all source populations are sampled (direct likelihood L_home). We simulated 10,000 individuals with Monte Carlo resampling, following the algorithm of Paetkau et al. (2004) and the frequencies-based method (Paetkau et al. 1995). The probability of type I error was set to *P* < 0.01, and the default frequency of missing alleles to 0.01.

## Results

Three hundred twenty-three individuals were successfully genotyped at 13 microsatellite loci, with the sample size for each island varying between 3 and 115 individuals (Table 1). All loci deviated significantly from HW equilibrium when all populations (Table 2) were analyzed together (Table 2). However, when tests were carried out within each population separately, only the genotypes frequencies at the AV3 locus consistently deviated from expectations at HW equilibrium. All results regarding genetic differentiation were similar when this locus was removed from the analyses; we therefore chose to present analyses where this locus was included. Departure from HW equilibrium was also detected over all loci on island 5 at Sleneset (Table 1), but not on any of the other islands. The contrasting results when HW was tested within each population and when all populations were pooled strongly suggest genetic differentiation between, but not within the populations. Fifty-nine of 234 pairs of loci compared within populations with at least 20 individuals (i.e. populations 5, 9, and 10) showed significant linkage disequilibrium (LD) after sequential Bonferroni correction. However, none of the loci showed consistent LD across all populations. We did not find any evidence for scoring error due to stuttering, large allele dropout or null alleles. Observed heterozygosity within the 14 populations ranged from 0.132 to 0.577 (Table 1). The number of alleles per locus ranged between 2 and 13 (Table 2). Allelic richness corrected for minimum sample size within each population ranged from 1.3 to 2.3, and although the inbreeding coefficients (*F*_IS_) were both negative and positive, none differed significantly from zero when correcting the *P*-value for the number of simultaneous tests (Table 1).

**Table 2:** Descriptive statistics of the 13 microsatellites used in 14 water vole populations in northern Norway when individuals from all populations where pooled; *N* number of individuals genotyped, *N*_A_ number of alleles, *H*_O_ mean observed heterozygosity, *H*_E_ mean expected heterozygosity, HW level of significance (*P*) for test of deviance from Hardy–Weinberg equilibrium

Locus	*N*	*N*_A_	*H*_O_	*H*_E_	HW
AV10	322	8	0.438	0.629	0.000
AV8	322	6	0.525	0.721	0.000
AV9	322	6	0.161	0.329	0.000
AV11	320	13	0.606	0.726	0.000
AV12	322	8	0.488	0.687	0.000
AV14	322	7	0.565	0.668	0.000
AV3	322	6	0.494	0.773	0.000
AT24	322	2	0.258	0.385	0.000
AT2	322	4	0.382	0.647	0.000
AT13	322	6	0.277	0.620	0.000
AT22	322	3	0.311	0.489	0.000
AT9	321	6	0.268	0.301	0.000
AT25	322	5	0.220	0.509	0.000

**Figure 3:**
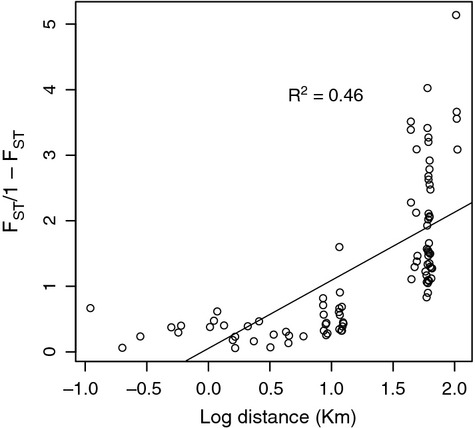
Isolation-by-distance analysis for water vole populations in northern Norway. The graph shows genetic distance (*F*_ST_* _=_ *F*_ST_/(1−*F*_ST_)) based on 13 microsatellite loci versus log-transformed geographic distance (kilometers) for all possible pairwise combinations among 14 water vole populations at the coast of northern Norway (2006).

**Figure 4:**
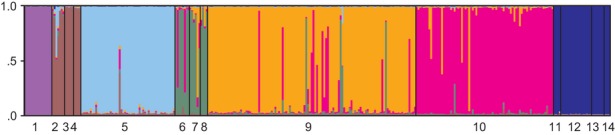
Results of Bayesian clustering of 323 water voles from 14 island populations within 4 localities in northern Norway with the program STRUCTURE. Population assignment to seven clusters is shown as colors on the bars representing the individuals. Numbers refer to the sampled islands in Table 1 and Fig. 2.

The overall level of genetic differentiation (measured by *F*_ST_) among populations was high (*F*_ST_ = 0.396 ± 0.042 (SE)). Accordingly, there was strong genetic differentiation between nearly all pairs of populations (Fig. 3), with *F*_ST_ ranging from 0.14 to 0.84, except for island pairs 13–14 and 12–14 at Lånan (Appendix 1). The genetic differentiation between populations increased with the geographic distance between them (Mantel *r* = 0.292, *P* = 0.0001, Fig. 3). The HIERFSTAT analysis showed that there was strong genetic differentiation both at the level of islands within localities (*F*_island/localities_ = 0.182, *P* < 0.0001) and at the level of localities (*F*_localities/total_ = 0.345, *P* < 0.0001). Moreover, according to the clustering analysis using both the method of Pritchard and Wen (2003) and the Evanno index (Evanno et al. 2005), individuals were grouped into 7 distinct clusters (Fig. 4, Appendix 2). One cluster corresponded to Myken; one included only individuals from Lånan and the other 5 included individuals from different islands in Sleneset and Lovund. Geneclass revealed the presence of 3 immigrants among islands within Lovund and Sleneset localities. One immigrant was detected between clusters 2 (made by islands 2, 3, 4) and 3 (island 5) at Lovund (Fig. 4), and two were detected among cluster 5 (island 9)and cluster 4 (islands 6, 7, and 8, Fig. 4). The immigrants were born in the same year of capture as determined by their body size and mass.

## Discussion

We found a high level of genetic differentiation between island populations of water vole on islands off the coast of northern Norway. This result is consistent with a low level of gene flow among islands. Accordingly, only 1% of all sampled and genotyped individuals were identified as immigrants.

Although comparison with the studies of Telfer et al. (2003) and Aars et al. (2006) is not straightforward because our microsatellite panels do no completely overlap, the level of genetic variation within populations (i.e. allelic richness and observed heterozygosity per locus) was similar to levels observed in island populations of water vole in Scotland (Telfer et al. 2003). Allelic richness was, on the other hand, much lower than what was found by Aars et al. (2006) in water voles living in both fragmented and continuous populations on the mainland (in UK and Finland). However, because the minimum sample size affects allelic richness, the comparison should be restricted to the islands with sample size larger than 45 (the minimum sample size in Aars et al. 2006), such as islands number 5, 9, and 10. When estimated for these three islands only, allelic richness ranged from 2.54 to 3.00. This suggests that each population included in our study might have been founded by few individuals and that genetic drift has also been reducing polymorphism within each island population. Across the study area, we found a rather strong spatial genetic structure, and geographic distance seems to be important for this pattern as distance explained a significant amount of variation in genetic differentiation among the sampled populations of water vole.

We found departure from HW equilibrium across all loci at one island and for one locus within all populations. Departure from HW equilibrium in water vole has been found also by Stewart et al. (1999) and Aars et al. (2006) and was in those cases attributed to the presence of both adults and young juveniles in the sample, which might also be the explanation in our study. However, in our case, deviation from HW equilibrium was found only in one of the 13 loci, which might suggest instead that mutations in the primer sequence(s) and the presence of non-amplifying alleles could be a possible explanation. This is, however, unlikely as the analysis with Microchecker did not indicate the presence of either scoring error due to stuttering, large allele dropout or any null alleles. Linkage disequilibrium has also been shown earlier to be a common feature for island populations of water vole (Stewart et al. 1999) and can be attributed to each population originating from few adult pairs in spring.

The inbreeding coefficients *F*_IS_ were generally low, and on several islands, the inbreeding coefficients were negative. Although none of the *F*_IS_ were significantly different from zero when multiple tests were accounted for, this is opposite to what is expected in small populations if inbreeding is common. However, negative *F*_IS_ are expected when there is outbreeding in small populations because of random differences in female and male allele frequencies (Rasmussen 1979; Luikart and Cornuet 1999). Furthermore, if dispersal is highly sex-biased, the probability of mating with a partner from a different area will be higher than when both sexes disperse (Prout 1981). Highly sex-biased dispersal is therefore expected to further decrease estimates of *F*_IS._ We identified too few dispersing individuals (*N* = 3) to test statistically if their sex ratio was biased toward one sex. Dispersal was detected only within the Sleneset and Lovund localities. However, it is likely that we underestimated dispersal because we did not sample all the island populations at each locality i.e. not all possible sources of immigrants have been assessed. Moreover, for example, at Lånan, the island populations were grouped by STRUCTURE into a single cluster, making it impossible to detect immigrants among them. The 7 distinct population clusters reflected the geographic structure of the populations. Moreover, the analysis with HIERFSTAT identified not only a strong level of genetic differentiation between localities, but also strong genetic differentiation between islands nested within localities. The localities Myken and Lånan are quite distant from the others (44–104 km and 59–104 km, respectively). But even Lovund and Sleneset, which are not so far apart (ca. 9 km), were identified as separate clusters and no immigrants were detected between them. The age of the identified immigrants suggests that dispersal occurs at juvenile stage in water voles, before reproduction has occurred. This is similar to what has been found based on capture-mark-recapture data (Lambin et al. 2004). Previous studies comparing continuous and highly fragmented environments failed to reveal a loss of genetic variability in metapopulations of both American pikas (Peacock and Ray 2001) and water voles (Aars et al. 2006). This questions whether fragmentation is actually reducing effective population size in small mammals and might make it difficult to detect metapopulation processes through genetic analyses (Lambin et al. 2004).

In Norway, water voles are perceived as pests and their conservation is currently not a concern (Bevanger 2007). However, a reduction in water vole population would have negative consequences for the ecosystem of which they are part. Coastal northern Norway is experiencing a growing developmental demand. For example, at Sleneset, there is a plan to build a windmill park, which, together with the related infrastructures, might seriously threaten the survival of the water vole populations. The new infrastructures might also favor the spread of alien predators, such as the American mink, potentially causing a drastic reduction in the water vole population. This would in turn have serious negative consequences for the only native water vole predator i.e. the endangered eagle owl. Our results strongly suggest small spatial scaling of evolutionary and population dynamic processes in water voles at the coast of Norway. On the basis of these results, we propose that conservation of water vole along the coast of Northern Norway should encompass as many island populations as possible.

## Appendix 1

Pairwise *F*_ST_ values (lower semi matrix) and pairwise geographic distances (upper semi matrix, in km) for 14 water vole populations along the coast of northern Norway.

**Table:**

Island nr.	1	2	3	4	5	6	7	8	9	10	11	12	13	14
1		44.19	44.11	44.08	44.59	49.88	48.71	49.17	49.32	47.31	102.84	105.41	103.93	104.03
2	0.69		0.60	0.50	0.57	8.97	9.05	12.13	12.01	12.16	59.91	62.42	61.06	61.18
3	0.78	0.29		0.11	1.11	8.54	8.56	11.59	11.48	11.58	60.19	62.69	61.33	61.46
4	0.77	0.27	0.40		1.03	8.64	8.66	11.70	11.59	11.69	60.18	62.68	61.32	61.45
5	0.53	0.23	0.32	0.27		9.09	9.27	12.41	12.28	12.52	59.42	61.93	60.56	60.68
6	0.59	0.30	0.45	0.36	0.31		1.58	4.52	4.27	5.92	57.95	60.34	59.12	59.26
7	0.68	0.20	0.42	0.24	0.22	0.15		3.39	3.19	4.47	59.52	61.91	60.69	60.84
8	0.76	0.41	0.62	0.48	0.31	0.20	0.21		0.28	2.09	61.31	63.65	62.49	62.64
9	0.58	0.24	0.38	0.26	0.28	0.23	0.06	0.19		2.34	61.04	63.38	62.22	62.37
10	0.56	0.24	0.40	0.36	0.30	0.19	0.12	0.28	0.14		63.32	65.67	64.50	64.65
11	0.84	0.66	0.80	0.77	0.52	0.55	0.61	0.77	0.60	0.56		2.58	1.18	1.34
12	0.76	0.67	0.74	0.72	0.52	0.57	0.62	0.71	0.60	0.56	0.32		1.65	1.65
13	0.79	0.67	0.76	0.73	0.51	0.54	0.61	0.74	0.60	0.56	0.38	0.19		0.20
14	0.78	0.59	0.72	0.68	0.47	0.45	0.52	0.67	0.57	0.53	0.29	0.05	0.06	

## Appendix 2

Graphical method to identify the true K (Evanno et al. 2005) from STRUCTURE analyses. Mean L(*K*) (± SD) of the posterior probability of the data for a given *K* over 5 runs of each *K* (left plot), and Δ*K*, the standardized second order rate of change of L(*K*) (right plot). The log likelihood increased with increasing *K*. Δ*K* showed the highest peak at Δ*K* = 7.
